# From Bench to Piglet: A Comparison of In Vivo and In Vitro Effects of Phytogenics on Post-Weaning Diarrhea, Growth Performance, and Bacterial Behavior

**DOI:** 10.3390/ani15111661

**Published:** 2025-06-04

**Authors:** Anika Weitmann, Sonja Axmann, Klaus Männer, Teemu Rinttilä, Tobias Aumiller

**Affiliations:** 1 Institute for Animal Nutrition and Feed, AGES GmbH—Austrian Agency for Health and Food Safety, 4020 Linz, Austria; anika.weitmann@ages.at (A.W.);; 2 Institute of Animal Nutrition, Department of Veterinary Medicine, Freie Universität Berlin, 14195 Berlin, Germany; 3 Alimetrics Research Ltd., Koskelontie 19B, FIN-02920 Espoo, Finland; 4 Delacon Biotechnik GmbH, 4209 Engerwitzdorf, Austria

**Keywords:** plant extracts, swine, biofilm, antimicrobial growth promoters, in vitro testing

## Abstract

Finding effective alternatives to antibiotics and zinc oxide in livestock feed remains challenging, but phytogenic compounds show promising potential. In the first part of the present study, the in vitro antimicrobial activities of carvacrol, eugenol, garlic oil, star anise oil, and tea tree oil as well as their effects on the biofilm formation of two *Escherichia coli* field isolates, quorum sensing of *Chromobacterium violaceum,* and the adhesion of an *E. coli* field isolate to piglets’ small intestinal mucus were determined. Based on these results, two prototypes were formulated. Phytogenic feed additive (PFA) Core 2, containing carvacrol, eugenol, and star anise oil, showed stronger in vitro antimicrobial activity, inhibition of biofilm formation, and quorum sensing than PFA Core 1, which was mainly composed of garlic oil and tea tree oil. In the second part of the present study, 1000 post-weaning piglets were divided into four groups receiving a control or diets with either PFA Core 1, PFA Core 2, or zinc oxide. Only PFA Core 2 and zinc oxide significantly improved body weight, daily gain, feed efficiency, and fecal scores compared with the control, while PFA Core 1 increased the feed efficiency and fecal scores. The results show that feed additives based on carvacrol and eugenol can improve the growth performance of post-weaning piglets and reduce the incidence of diarrhea, possibly by influencing detrimental bacteria. Furthermore, the present study demonstrates the potential of combinations of in vitro assays to support the development of effective feed additives.

## Simple Summary

The post-weaning period in piglet production is associated with bacterial disorders and challenges such as diarrhea. Bioactive plant compounds are promising alternatives to traditional antimicrobial growth promoters, although translating lab studies to field conditions remains challenging. This study investigates the antimicrobial properties of bioactive plant compounds to tackle these issues. Carvacrol, eugenol, garlic oil, star anise oil, and tea tree oil were screened for their antimicrobial activities, effects on biofilm formation, bacterial communication, and adhesion to piglet intestinal mucus. Based on the results, two prototypes were created. Prototype two, containing carvacrol, eugenol, and star anise oil, showed stronger antimicrobial activity and better inhibition of biofilm formation and bacterial communication than prototype one, which contained garlic oil and tea tree oil. In part two of the study, 1000 post-weaning piglets were divided into four groups: the control group and three treatment groups receiving diets with prototype one, prototype two, or zinc oxide. Prototype two and zinc oxide improved body weight, daily gain, feed efficiency, and fecal scores compared to the control. The results of this study suggest that the compounds in prototype two can support piglets by likely influencing intestinal bacteria and demonstrate the potential of combining lab tests to develop effective feed additives.

## 1. Introduction

For decades, antibiotics have been used by the livestock industry as in-feed antimicrobial growth promoters (AGPs). But rising concerns regarding the development of bacterial resistance to clinically important antibiotics have intensified the efforts to reduce their routine application in recent years. In this context, several countries have already banned the use of antibiotics as antimicrobial growth promoters [1], including the European Union in 2006 [2]. Because of this ban, zinc oxide (ZnO) was used as a replacement throughout Europe, especially in pig feed, to improve the animal’s growth performance and reduce the incidence of post-weaning diarrhea (PWD). As a growth promotor, ZnO is applied in pharmacological doses of 2000 to 4000 ppm, resulting in a correspondingly high accumulation in manure and the environment [3]. Therefore, the EU has restricted the application of ZnO beginning in 2022 [4]. These regulatory provisions, combined with an increasing pressure from consumers demanding more “natural” solutions, necessitates the search for alternatives to traditional AGPs in order to maintain efficient farming in the future. Especially promising in this regard are plant-derived bioactive compounds (hereinafter referred to as phytogenics).

Phytogenics are widely used in swine diets to influence palatability. Phytogenic flavors such as anise and garlic have been shown to positively impact feed intake in piglets [5]. Many phytogenics are also known to have bactericidal effects [6]. Furthermore, specific phytogenics have been shown to affect bacterial behavior and pathogenicity at concentrations below the minimum inhibitory concentration (sub-MICs) [7,8,9]. Several of these effects are attributed to the ability of specific phytogenics to interfere with the bacterial communication system quorum sensing [9]. Quorum sensing is based on the detection of secreted signal molecules, which accumulate in the environment in a cell density-dependent manner and are involved in the regulation of a wide variety of bacterial behaviors including bioluminescence, motility, virulence factor expression, biofilm formation, and pigment production [10,11,12,13,14]. Phytogenics that have been shown to influence quorum sensing include single substances like carvacrol [8], eugenol [15], or trans-anethole [16], and essential oils such as garlic oil [17] or tea tree oil [7]. Besides their various effects on pure bacterial cultures, challenge studies on the model organism *Caenorhabditis elegans* [18,19] and human cell lines [20] have demonstrated that phytogenics can also interfere with the quorum sensing of pathogenic bacteria in these models, leading to attenuated colonization and increased survival. Therefore, it is assumed that the application of phytogenics as feed additives in livestock could reduce the expression of bacterial virulence factors by interfering with quorum sensing signaling. This would likely prevent or reduce the burden of bacterial infections on the animal host while minimizing the selective pressure to develop resistance against the applied compound or treatment [21,22]. Livestock animals could benefit from these effects particularly during critical phases of their development, such as piglets in the post-weaning period [3].

The development of phytogenics-based feed additives for livestock animals, however, faces major challenges since severe limitations exist in terms of in vivo trial capacities as it is practically not feasible to test compounds in studies covering detailed dose–response relationships and possible combinations of different phytogenics. The sheer number of animals such trials would require contradicts the ethical responsibility and the “Replacement, Reduction, and Refinement” (3Rs) framework. The 3Rs aim to minimize the reliance on animal trials while maintaining robust scientific rigor [23]. Addressing these challenges requires alternative approaches to livestock feeding trials, such as leveraging advanced in vitro models and computational simulations.

Taking the above considerations into account and recognizing the limitations, the present study investigated the impact of carvacrol, eugenol, garlic oil, star anise oil, and tea tree oil as individual substances and as main constituents of two phytogenic blends as well as ZnO as a reference compound on three different bacterial models for quorum sensing-related traits. Subsequently, the two phytogenic blends were used as feed additives and studied in a feeding trial to evaluate their in vivo potential to reduce the incidence of PWD and improve the growth performance of piglets during the critical post-weaning phase in comparison to ZnO. Therefore, the hypothesis in the present study is that these two prototypes, developed based on the consistent effects of phytogenic compounds observed in a series of in vitro assays, will demonstrate distinct and corresponding responses in the piglet model. This will help enhance the understanding of how these models can predict in vivo effects.

## 2. Materials and Methods

### 2.1. Bacterial Strains and Test Compounds

*Chromobacterium violaceum* DSM 30191 was purchased from the German Collection of Microorganisms and Cell Cultures (Braunschweig, Germany) and cultured in trypto-casein soy broth (Biokar Diagnostics, Allone, France; TSB) at 30 °C under aerobic conditions.

*Escherichia coli* field isolates O88:H8 and O143:H4 (both isolated from swine) were provided by the veterinary pathology department of AGES GmbH Linz (Linz, Austria) and serotyped by the national reference laboratory for *E. coli* of AGES GmbH Graz (Graz, Austria). Both strains were classified as pathogenic due to their high-level growth in pure culture in various organs and their mucoid or hemolytic properties. For the mucus adherence assay, a F4-fimbriated *E. coli* strain isolated in Finland was obtained as a field isolate by Alimetrics Research Ltd. (Espoo, Finland). The *E. coli* field isolates were cultured under aerobic conditions at 37 °C in TSB that was supplemented with 2 g/L glucose (Carl Roth GmbH + Co. KG, Karlsruhe, Germany; TSB^+^) for biofilm assays.

Zinc oxide for the bacterial assays was purchased by Auhof Apotheke (Linz, Austria). Carvacrol, eugenol, garlic oil, star anise oil, and tea tree oil as well as “PFA Core 1” and “PFA Core 2” were provided by Delacon Biotechnik GmbH (Engerwitzdorf, Austria). “PFA Core 1” contained 65.8% tea tree oil, 32.8% garlic oil, and 1.4% citrus oil. “PFA Core 2” was composed of 40% carvacrol, 40% eugenol, and 20% star anise oil. Both essential oil cores were mixed with a carrier matrix. For individual essential oils, the main compounds are trans-anethole for star anise oil, terpinene-4-ol and γ-terpinene for tea tree oil, diallyl disulfide and diallyl trisulfide for garlic oil, and D-limonene for citrus oil.

### 2.2. Determination of Minimum Inhibitory Concentrations

The minimum inhibitory concentration (MIC) in each assay was optically determined as the lowest concentration of the test compound that completely inhibits visible growth (no obvious cell pellet or turbidity) after the incubation period (18 h at 30 °C for *C. violaceum*, 18 h at 37 °C for *E. coli* O88:H8 and O143:H4, and 24 h at 37 °C for the F4-fimbriated *E. coli* strain). The median value of all replicates was defined as the MIC value of the respective test compound.

The F4-fimbriated *E. coli* strain was only tested with carvacrol and tea tree oil. For MIC evaluation, both substances were diluted in a two-fold series in TSB containing 2% Tween 80 (Sigma-Aldrich, St. Louis, MO, USA).

### 2.3. Quorum Sensing Inhibition Assay (Chromobacterium violaceum)

The influence of the phytogenic test compounds on *C. violaceum* was assessed in concentrations ranging from 10,000 ppm to 5 ppm. Violacein production was quantified based on the protocol of Skogman et al. [24]. Serial dilutions ranging from 20,000 ppm to 10 ppm were prepared from stock solutions (200,000 ppm) of the phytogenic test compounds in ≥99.8% ethanol (VWR International GmbH, Vienna, Austria). Four technical replicates of each concentration (50 µL in each well) were arranged vertically in Nunc^®^ polystyrene 96-well microtiter plates (Thermo Scientific, Waltham, MA, USA). An overnight culture of *C. violaceum* in 10 mL TSB was diluted to approximately 1–2 × 10^6^ CFU/mL in fresh TSB, and aliquots of 50 µL were added to each test well and four wells containing only medium, serving as positive growth control without test compounds. An additional four wells containing only TSB were used as negative (sterility) controls. The starting bacterial concentration was verified using the colony count method. The microtiter plates as well as the colony count plates were incubated for 18 h at 30 °C. After the incubation period, MIC values were determined before the cells and violacein pigment were pelleted by centrifugation at 3000 rpm for 10 min. The supernatant was removed from the wells by pipetting and 200 µL ≥ 99.8% ethanol (VWR International GmbH, Vienna, Austria) was added to each well. The plates were sealed with Microseal ‘B’ PCR Plate Sealing Film (Bio-Rad, Hercules, CA, USA) and incubated overnight in the dark to allow the pelleted violacein pigment to dissolve. The next day, the plates were centrifuged at 3000 rpm for 10 min to sediment the decolored cells. Subsequently, 100 µL of the violacein-stained supernatant was transferred to a new microtiter plate and the absorbance was measured at 570 nm using a PHOmo microplate reader (Anthos Mikrosysteme GmbH, Friesoythe, Germany).

### 2.4. Biofilm Inhibition Assay

The effect of the phytogenic test compounds on the biofilm formation of *E. coli* O88:H8 and O143:H4 was assessed in a broth micro-dilution assay followed by crystal violet staining according to Axmann et al. [25], with minor modifications. Briefly, phytogenic test compounds were serially diluted in TSB^+^ and arranged vertically in 96-well microtiter plates, as described in Section 2.3. The outer columns of the microtiter plates were filled with sterile TSB^+^ to prevent evaporation from the central wells. Overnight cultures of the respective *E. coli* in TSB^+^ were adjusted to approximately 2 × 10^6^ CFU/mL in fresh TSB^+^ and 50 µL of this dilution was placed in each test well. Four wells containing either only bacterial culture without phytogenic test compounds or sterile TSB^+^ medium served as positive or negative control, respectively. The plates were incubated under aerobic conditions for 18 h at 37 °C.

The antimicrobial activity of the phytogenic test compounds was evaluated as described in Section 2.2. After removal of the culture medium, the microtiter plates were washed twice with deionized H_2_O (diH_2_O) to remove the unattached cells and media components and air-dried for 30 min. The attached biomass was stained with 130 µL of a 0.1% (*w*/*v*) crystal violet solution (Alfa Aesar by Thermo Fisher GmbH, Kandel, Germany) in diH_2_O for 20 min at room temperature. The plates were then washed three times with diH_2_O and left to air-dry completely. The adherent dye was solubilized with 130 µL 30% (*v*/*v*) acetic acid (Merck KgaA, Darmstadt, Germany) and transferred to a new microtiter plate. Absorbance was measured at 570 nm with a PHOmo microplate reader (Anthos Mikrosysteme GmbH, Friesoythe, Germany). The mean of the four replicates was calculated followed by subtraction of the negative control measurements, and the results were expressed as a percentage biofilm relative to the positive control. Each test compound was assayed three to four times. In this study, the entirety of adherent cell mass that was stained by the crystal violet solution is referred to as biofilm without distinction of the maturation stage.

### 2.5. In Vitro Mucus Adherence Assay

In preparation of the mucus adhesion assay, an overnight culture of F4-fimbriated *E. coli* was grown at 37 °C in TSB without test compounds. From this seed culture, two successive overnight cultivations were performed for each treatment using 10% inoculum. This assay was only performed with carvacrol and tea tree oil in concentrations of 1/8th of the MIC (320 ppm for tea tree oil and 20 ppm for carvacrol). In addition, a second concentration of tea tree oil at 1/80th of the MIC was assayed to achieve a concentration level comparable to the tested carvacrol concentration. All treatments were cultured at 37 °C in three replicate vials. The turbidity of the cultures was followed by measuring the absorbance at 600 nm to ensure that the cell densities were close to those of the control culture without a test product. On the third day, each culture was further diluted tenfold in growth medium containing the respective test product at its specific concentration. Tritium-labeled thymidine was introduced in each vial to radioactively label the *E. coli* cells. For two hours, the culture was incubated at 37 °C. During incubation, the bacteria were taking up the radioactive nucleotide and incorporated it in their DNA. The radioactive-labeled bacteria were harvested by gentle centrifugation (3000× *g* for 5 min) and re-suspended in an equal volume of HEPES-Hank’s buffer (Nordic Biosite, Täby, Sweden). The suspension was used immediately for the adherence study.

For the in vitro adherence study, authentic mucus samples were collected from five piglets, pooled, and aliquoted. The aliquots were stored at −80 °C until further use in the study. To prepare mucus plates, the mucus samples were diluted with a coating buffer and cleared off all insoluble particles such as epithelial cells and bacteria by high-speed centrifugation. Mucus preparations were diluted to the final protein concentration of 0.1 mg/mL. These preparations were then used to coat microtiter wells that bind mucus irreversibly. The coated microtiter plates were washed twice with 300 mL of HEPES-Hank’s buffer to wash off the unbound mucus components before labeled *E. coli* cells were introduced (nine replicate wells per treatment) and incubated for 1 h at 37 °C. The reaction liquid was discarded and the wells were washed twice with 300 μL of HEPES-Hank’s buffer to remove unbound radioactive bacteria. Then, 250 μL of scintillation liquid was added and the radioactivity in each reaction well was measured. Additionally, the total radioactivity of each labeled *E. coli* culture used in the adherence study was measured to be able to determine the proportion of adhered cells. Hence, the remaining radioactivity in the wells was proportional to the number of adhered pathogenic bacteria.

### 2.6. Animals, Diets, and In Vivo Trial Design

A total of one thousand healthy post-weaning piglets (500 ♂; 500 ♀; DanBred × Duroc), with an age at weaning of 25 ± 2 days, were allocated to four treatments and 100 pens with solid partitions and slotted floor in total (5 ♂ & 5 ♀ per pen, 25 pens per treatment). The average housing temperature was kept at about 30 °C during the first week after weaning and was gradually reduced by 1.8 °C per week down to 22 °C from day 53 of age onwards. Post-weaning piglets had ad libitum access to feed provided in mash form and water supplied by drinking bowls throughout the experiment. Prior to weaning, piglets had access to creep feed formulated without added antibiotics, organic acids, polysaccharides, enzymes, yeast/egg products, porcine plasma, or zootechnical feed additives. The concentrations of zinc and copper were maintained at adequate (nutritional) levels, but not in excess, to avoid the potentially confounding effects of these additives.

The four dietary treatments included a negative control group receiving an unsupplemented basal diet (negative control; NC), a positive control group whose diet was supplemented with ZnO at a pharmacological level (3 kg/t of feed; ZnO), and the treatment groups PFA 1 and PFA 2 that were fed basal diets supplemented with PFA 1 or PFA 2, respectively, at 1 kg/t of feed each.

Phytogenic prototype PFA 1 consisted of wheat bran, limestone, and a blend of turmeric and fenugreek seed powder, with the addition of PFA Core 1. Phytogenic prototype PFA 2 was composed of limestone, a blend of turmeric and fenugreek seed powder, and PFA Core 2. Both PFA cores were added at the same inclusion level of 50 g/kg to the respective prototype.

The 42-day observation period was divided into two feeding periods: a starter period of two weeks (from 25 to 38 days of age) and a subsequent grower period of four weeks (from 39 to 66 days of age). The basal diets for each period were formulated as recommended by the Society of Nutrition Physiology [26], with the exception of zinc, and are presented in Table 1. Delacon Biotechnik GmbH (Austria) supplied the phytogenic prototypes. Zinc oxide (Spezialfutter Neuruppin GmbH & Co. KG, Neuruppin, Germany) was supplemented at the expense of Tixosil (>97% silicon dioxide), while PFA 1 and PFA 2 were included at the expense of wheat bran. This substitution was made to leverage the prebiotic potential of wheat bran and the carrier components of the phytogenics, in contrast to the nutritionally inert nature of Tixosil and ZnO.

### 2.7. Piglet Growth Performance and Determination of Diarrhea Score

All piglets were observed twice daily for any abnormalities, abnormal behavior, and clinical signs of sickness throughout the 42 d experimental period.

Pen body weights (BWs) were recorded weekly, as was the amount of feed supplied to each pen during the preceding week. Piglet BW gain was calculated by dividing the mean BW per pen at the end of each period by the mean BW per pen at the start of each period and number of piglets per pen. Feed consumption per piglet was estimated as the total feed supplied per pen and period, corrected for dispersed/leftover feed and the number of piglets per pen. The feed-to-gain ratio (FCR) was calculated from the relationship of the weekly corrected feed intake and BW gain per piglet for this period. The diarrhea scores were determined on a pen-basis using a scale from 0 to 3 (0 = normal; 1 = soft feces; 2 = mild diarrhea; 3 = severe diarrhea).

### 2.8. Statistical Analysis

**Figure 1:**
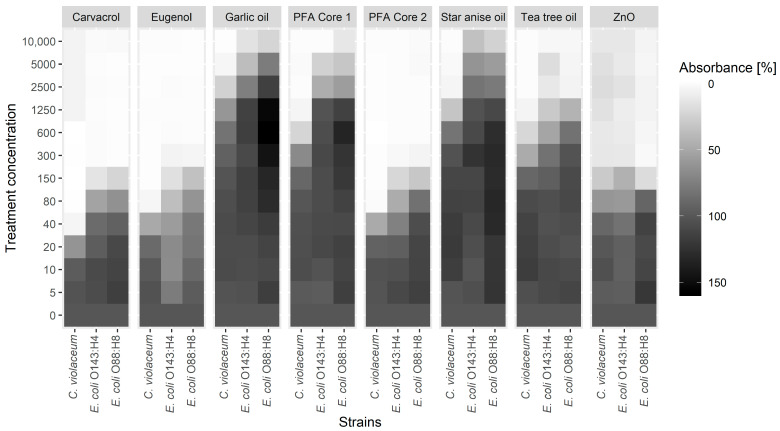
Impact of carvacrol, eugenol, garlic oil, star anise oil, tea tree oil, phytogenic feed additive (PFA) Core 1, PFA Core 2, and ZnO on violacein production of *C. violaceum* and biofilm formation of *E. coli* strains O143:H4 and O88:H8. Absorbance values for each concentration were normalized to the respective positive control (containing no phytogenic compound).

*Chromobacterium violaceum* violacein production and *E. coli* biofilm formation was analyzed using the Glimmix procedure of SAS (SAS 9.4, SAS Institute Inc., Cary, NC, USA), with substance concentration as a fixed effect and the day of measurement (biological replicate) as a random effect. Data from the mucus adherence assay were analyzed with the Student’s *t*-test. A comprehensive overview of the *C. violaceum* and *E. coli* results is given in Figure 1, with a detailed presentation of the results supplied in Supplementary Figures S1–S8.

The in vivo study design was a random complete block design, with weaning batch as the blocking factor and pen as the experimental unit for statistical purposes for recoded parameters. Growth performance was analyzed using the Glimmix procedure of SAS. Treatment was used as a fixed effect and the results were presented as the least square means with standard error of the mean. Body weight day 1 was used as the co-factor for analysis of BW at day 15, BW at day 42, average daily gain (ADG), and average daily feed intake (ADFI).

Fecal consistency was assessed using a generalized linear mixed model (GLMM) with binomial distribution, fitted via the glmer function in R (v4.1.2). The original 4-point fecal score (0 = normal; 1 = soft feces; 2 = mild diarrhea; 3 = severe diarrhea) was recorded into a binary outcome: 0 = normal feces, 1 = altered feces. The model included the treatment, period, as well as their interaction as fixed effects, and weaning batch as a random effect. Type III tests with Kenward–Roger approximation were performed using the car package.

## 3. Results

### 3.1. Effect of Selected Phytogenics on Bacterial Growth, Violacein Production, and Biofilm Formation

In the present study, the influence of ZnO, two phytogenic prototype essential oil cores (PFA Core 1 and PFA Core 2), and their respective main compounds garlic oil and tea tree oil, as well as carvacrol, eugenol, and star anise oil, was initially investigated on bacterial susceptibility, biofilm formation of *E. coli* strains O88:H8 and O143:H4, and violacein production of *C. violaceum*. Treatment with each substance exerted a similar effect on the growth of all bacterial strains (Table 2). In particular, carvacrol and eugenol showed very strong bactericidal effects (MIC of 150–600 ppm), whereas tea tree oil exhibited substantial lower antimicrobial activities against all three *E. coli* strains (MIC of 2560–10,000 ppm) and *C. violaceum* (MIC of 2500 ppm). Similarly, garlic oil and star anise oil only inhibited the growth of *C. violaceum* at 10,000 and 5000 ppm, respectively, while the growth of both tested *E. coli* strains was not inhibited. Zinc oxide did not inhibit the growth of the *E. coli* and *C. violaceum* strains up to the highest tested concentration of 10,000 ppm. The MIC values of substance combinations PFA Core 1 and PFA Core 2 were similar to those of the respective individual substances.

The observed values for biofilm formation and violacein production at each treatment concentration were compared to an untreated positive control to identify potential sub-MIC effects. Both parameters decreased in a dose-dependent manner at sub-MIC levels in response to each tested substance (Figure 1 and Figures S1–S8). Eugenol and carvacrol proved to be the most potent of the substances tested in reducing biofilm formation of the *E. coli* strains. Interestingly, although carvacrol showed identical effects on the biofilm formation of both *E. coli* strains, eugenol elicited a different response in the two strains, with a concentration of 10 ppm disturbing biofilm formation in *E. coli* O143:H4, while 80 ppm was required to do so in *E. coli* O88:H8. The biofilm-reducing effect of the carvacrol-eugenol-based PFA Core 2 was in the same range as that of the two individual substances with 80 ppm for *E. coli* O88:H8 and 40 ppm for *E. coli* O143:H4. In contrast, the third component of PFA Core 2, star anise oil, showed comparatively weak effects and required a minimum of 5000 ppm to achieve a significant response. Garlic oil did not show any significant effects below 5000 ppm on the biofilm formation of either strain. Tea tree oil significantly reduced the biofilm formation of both *E. coli* strains in three to four sub-MICs by −96.6% to −48.5%. Likewise, PFA Core 1 significantly decreased (−87.7% to −44%) the biofilm formation of *E. coli* O88:H8 and O143:H4 in two and three sub-MICs (down to 2500 ppm), respectively. Although ZnO did not inhibit the growth of either *E. coli* strain, the substance did show a strong impact on their biofilm formation capabilities, with the lowest effective concentration ranging from 150 ppm (−81.4%) for *E. coli* O88:H8 to 80 ppm (−41.7%) for *E. coli* O143:H4.

Violacein production of *C. violaceum* was most efficiently reduced with carvacrol, eugenol, and PFA Core 2, with the lowest effective concentration being 20 ppm. While tea tree oil and PFA Core 1 showed similar potentials to inhibit violacein production in the first three sub-MICs (−96.5% to −32.4% at 1250–300 ppm), garlic and star anise oil proved to be less effective, with a violacein-inhibiting concentration of 600 ppm and 300 ppm, respectively. Interestingly, low concentrations of tea tree oil (10 ppm) and star anise oil (10–150 ppm) slightly increased the violacein production of *C. violaceum*. Comparable to the observations on biofilm formation, ZnO decreased the violacein signal down to a concentration of 40 ppm, despite having no effect on the growth of *C. violaceum*.

### 3.2. Effect of Carvacrol and Tea Tree Oil on Adhesion of F4-Fimbriated E. coli to Piglets’ Small Intestinal Mucus In Vitro

**Figure 2:**
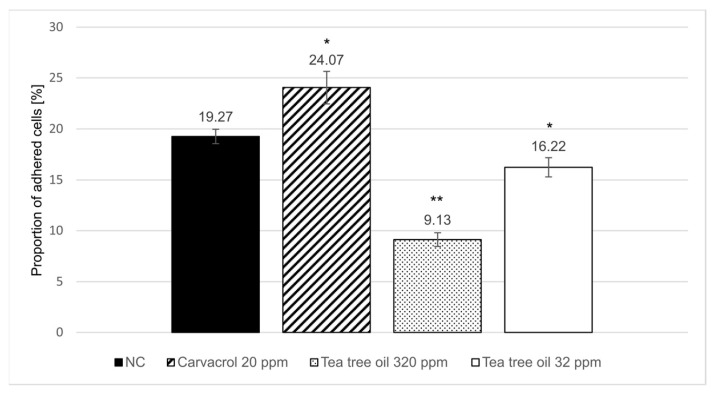
Mucus-bound F4-fimbriated *E. coli* presented as percentage of introduced labeled bacteria. The error bars indicate standard error of nine replicate wells and asterisks indicate the statistical difference in comparison to the negative control (NC) analyzed with a *t*-test (*~*p* < 0.05, **~*p* < 0.001).

The effect of carvacrol and tea tree oil on the mucus-binding properties of an F4-fimbriated *E. coli* strain was evaluated in an in vitro mucus adherence assay. Close to 20% of the radioactive-labeled F4-fimbriated *E. coli* introduced into the microtiter wells adhered to the mucus coating (Figure 2). This suggests that all receptor sites in the mucus became occupied by the introduced bacterial cells grown in the absence of the test products. Treatment with tea tree oil and carvacrol showed opposite effects on the adhesion of F4-fimbriated *E. coli* cells; exposure to tea tree oil reduced the proportion of adhered cells in both tested concentrations (−52.6% at 320 ppm and −15.8% at 32 ppm), while carvacrol enhanced bacterial adhesion by +24.9% at 20 ppm.

### 3.3. Effect of Phytogenic Prototypes on Piglet Growth Performance and Health Parameters

The in vivo study was run without any adverse technical events. Growth performance data are shown in Table 3. Piglets receiving a pharmacological dosage of 3 kg ZnO per ton of feed showed the best growth performance with a 12.56% improved ADG during the 42-day trial period, resulting in a 9.19% difference in BW at the end of the trial compared to the NC. The two phytogenic prototypes did not differ significantly in their effect on the piglets. However, in comparison to the NC, PFA 2 significantly improved ADG (7.05% from day 1 to day 42) and FCR (−4.4% from day 1 to day 42), which led to a 5.25% increased final BW at the end of the study. Additionally, dietary supplementation with PFA 2 caused ADFI to increase significantly during the first two weeks of the study compared to the NC. Supplementation with PFA 1, on the other hand, only improved FCR to a significant extent.

The mortality observed throughout the whole study period of 42 days was NC: 1.6%, ZnO: 0.4%, PFA 1: 0.8%, and PFA 2: 0.4% (Table S1). The medication rate amounted to NC: 6.4%, ZnO 5.6%, PFA 1: 7.2%, and PFA 2: 5.5% (Table S2). Clinical signs of PWD were found in 2.4% (6 animals) of piglets fed the unsupplemented basal diet (NC) and 1.2% (3 animals) or 0.8% (2 animals) of piglets fed the diet supplemented with PFA 1 or PFA 2, respectively. No piglets receiving ZnO were affected by clinical signs of PWD. Remaining piglets showed normal activity and alertness, a normal coat and eyes, as well as normal feces and urine.

The fecal scores were recorded based on a scale from 0 to 3 (0 = normal; 1 = soft feces; 2 = mild diarrhea; 3 = severe diarrhea) and are depicted in Table 4. Since no signs of severe diarrhea (scale 3) were observed during the in vivo study, fecal scores were binarized as 0 = normal and 1 = altered (soft to severe diarrhea). During the starter feeding period (day 1 to 14), zinc oxide supplementation (ZnO, treatment 2) resulted in the highest probability of normal feces (0.821), followed by PFA 2 (treatment 4; 0.681), PFA 1 (treatment 3; 0.579), and the negative control (NC, treatment 1; 0.422). In the grower feeding period (day 15 to 42), all supplemented treatments showed high probabilities of normal feces, with ZnO again being the highest (0.983), followed by PFA 2 (0.898), PFA 1 (0.855), and NC (0.870).

## 4. Discussion

Many challenges in livestock production are associated with bacterial infections. This is especially the case for critical phases early in the life of an animal. In this regard, piglets are among the most susceptible livestock species, particularly during the weaning period, with massive environmental, dietary, and social changes for these young animals. Stress and an increased susceptibility to health-issues such as diarrhea are the consequence. One of the most common strategies to manage PWD is the application of ZnO at pharmacological concentrations [3]. However, with increasing restrictions on the use of traditional AGPs like antibiotics and ZnO, new strategies are required to promote animal welfare and livestock production efficacy. Therefore, the present study assessed the potential of selected phytogenics as alternatives to ZnO, the interactions of these substances with different bacterial models, and their performance in a piglet feeding trial.

The microbroth dilution assay of the individual substances revealed a substantially stronger antibacterial efficacy of carvacrol and eugenol compared to tea tree oil, garlic oil, and star anise oil in all tested strains. This is well in line with available references in which carvacrol [25,27,28] and eugenol [28] proved to be potent substances to inhibit bacterial growth, while tea tree oil [7] and garlic oil [29,30] showed only weak antibacterial efficacy. The application of lethal concentrations of a substance could, however, be a strong inducer of resistance development. Consequently, it is of great interest to explore the potential of phytogenics to prevent the expression of pathogenic traits without exerting selective pressure towards resistance development or compromising the “healthy” functions of the microbiome. In this regard, the influence of phytogenics on the cell density-dependent bacterial communication system quorum sensing has gained considerable interest as many virulence-related genes are regulated by quorum sensing [21]. Therefore, several bacterial models related to quorum sensing were applied in this study to investigate the inhibitory potential of phytogenics.

At first, the biosensor strain *C. violaceum* was used as a model to determine the QS-controlled violacein production. The treatment of *C. violaceum* with all individual substances at sub-MIC concentrations inhibited or reduced the production of violacein (Figure 1). These results suggest that the tested substances may interfere with quorum sensing of this model strain, although at different concentrations. Similar results were obtained by Burt et al. [8] for carvacrol, Alvarez et al. [7] for tea tree oil, Noumi et al. [31] for star anise oil, and Zhou et al. [15] for eugenol, which reported an inhibition of violacein production in *C. violaceum* at concentrations comparable to those in the present study.

The literature references for the effect of garlic oil on *C. violaceum* are not available to the authors’ knowledge. However, Bodini et al. [32] found that aqueous garlic extracts inhibited the violacein production of two *C. violaceum* strains at similar concentrations as the garlic essential oil used in the present studies. Most studies investigating the effects of ZnO are using nanoparticles in antibacterial assays. For example, Al-Shabib et al. [33] and Khan et al. [34] used different types of ZnO nanoparticles and observed violacein reduction in *C. violaceum* biosensor strains beginning at 50 ppm and 25 ppm, respectively.

Quorum sensing has also been shown to be involved in the formation of biofilms [11,13]. The protective environment of biofilms plays an important role in bacterial infections by conferring resistance towards antibiotics and host defense systems [12,35]. In this study, carvacrol, eugenol, tea tree oil, and ZnO showed a significant impact on the biofilm formation of two *E. coli* field isolates at sub-MIC concentrations, although carvacrol, eugenol, and ZnO exhibited these effects at much lower concentrations than tea tree oil (Figure 1). Star anise oil did not have an MIC within the tested concentration range and only showed an effect on biofilm formation at concentrations of 5000 ppm or higher. Garlic oil, on the other hand, seemed to have weak biofilm-promoting properties on *E. coli* strain O88 at sub-MIC concentrations between 300 and 1250 ppm. The disturbance of *E. coli* biofilm production by carvacrol [25], eugenol [36], ZnO [33], and tea tree oil [37] was also found by previous studies in a similar concentration range, causing the effects in the present study. Despite the slightly stronger antimicrobial effects against *C. violaceum* compared with the two pathogenic *E. coli* field isolates, the phytogenic compounds decreased the violacein production, and hence the quorum sensing, of *C. violaceum* in a similar sub-MIC range than the biofilm formation of the *E. coli* field isolates. Therefore, it is hypothesized that the reduction of biofilm formation induced by carvacrol, eugenol, tea tree oil, and ZnO could be related to their disturbing influence on quorum sensing. Likewise, Burt et al. [8] found that carvacrol suppressed violacein production at concentrations consistent with carvacrol’s inhibiting effect on the biofilm formation of *C. violaceum*, *Salmonella enterica* subsp. Typhimurium, and *Staphylococcus aureus*. They concluded that carvacrol’s activity to inhibit biofilm formation may be related to the disruption of quorum sensing.

The third model applied in this study evaluated the impact of tea tree oil and carvacrol on the ability of an F4-fimbriated *E. coli* strain to adhere to the small intestinal mucus of piglets. This is especially relevant in the livestock industry, as F4-fimbriated *E. coli* are one of the major pathogens associated with PWD, and the production of F4 fimbriae is regulated by quorum sensing [14]. Previous studies demonstrated the potential of cranberry extract to inhibit the adherence of F4- and F18-fimbriated *E. coli* to intestinal villi in vitro and intestinal epithelium in vivo [38]. A subsequent challenge experiment revealed that cranberry supplementation of feed and drinking water reduced diarrhea as well as the extent and duration of F18-fimbriated *E. coli* excretion [38]. Thus, it was speculated that tea tree oil, which reduced the adhesion of F4-fimbriated *E. coli* in the present study, might also increase the resilience of piglets against PWD when supplemented in feed. Treatment with carvacrol, on the other hand, resulted in a higher number of adherent cells, which may indicate increased expression of adhesion factors. However, it is more likely that the chemical stress induced by carvacrol led to the occurrence of autoaggregation events. This type of bacterial behavior serves to protect the involved bacteria from external stresses [39]. However, further studies are necessary to determine the exact nature and cause of this observation.

Based on the results of the investigation of the individual phytogenic compounds, two prototypes were formulated, PFA Core 1 and PFA Core 2. PFA Core 1 was based on garlic oil and tea tree oil, while the second prototype, PFA Core 2, was based on carvacrol, eugenol, and star anise oil. The prototypes were formulated based on three different considerations: Due to their antibacterial efficacy and sub-MIC effects, carvacrol and eugenol were selected as the most potent substances from the present in vitro tests and used as the basis for one of the prototypes. The convincing results of tea tree oil in the mucus adherence assay led to the assumption that this substance could be promising in terms of increasing the resilience of piglets to PWD. Therefore, the second prototype was based on tea tree oil. In addition, both garlic oil and star anise oil were added to the prototypes due to their potential feed intake-stimulating properties, although the present results suggest the little relevance of both substances in terms of their influence on PWD caused by pathogenic *E. coli*. The subsequent study of the bactericidal activity as well as quorum sensing and the biofilm inhibitory properties of the two prototypes showed that the effects of both PFA cores correlated with those of their respective individual main components (Figure 1 and Table 2).

The in vitro test of ZnO, which served as a positive control in the in vivo feeding trial, revealed no bactericidal activity in the applied concentration range. Evidence from the literature suggests that particle size has a major influence on the antibacterial activity of ZnO [40]. ZnO nanoparticles especially showed stronger antibacterial effects than larger commercial particles, which were used in this study to mimic the application in livestock. Consequently, the high average particle size in combination with low water solubility might have been responsible for the lack of bactericidal activity of the pharmaceutical-grade ZnO applied in the present study. Despite this, a strong inhibition of *E. coli* biofilm formation and *C. violaceum* violacein production was observed in a wide range of tested ZnO concentrations. These findings indicate that ZnO may not only function through bactericidal effects but also inhibit bacterial traits in the sub-MIC range.

Side effects like the onset of toxic effects during prolonged administration and the accumulation of the heavy metal zinc in the environment prompted the European Union to ban the use of medicinal doses of ZnO, starting from June 2022 [3]. At the same time, the 3Rs principle is already firmly established in the EU legislation and requires scientists to carefully consider the 3Rs principle in their search for alternative feed additives [23]. Due to this background and the encouraging results of the in vitro assays, where the prototypes showed similar effects as the individual substances, only the prototypes were included in the subsequent in vivo trials. Therefore, the second part of the present study evaluated the in vivo potential of the formulated phytogenic feed additive prototypes as ZnO alternatives in piglets during the nursery period. While feed additives containing carvacrol have already been employed in previous studies, the use of tea tree oil in weaning piglets is much less common.

The supplementation of feed with ZnO resulted in the expected improvement in the growth performance of the animals along with a significant reduction in fecal scores. Similar benefits of ZnO on piglet growth parameters and fecal scores were reported by other studies. For instance, Molist et al. [41] observed a reduced incidence of diarrhea and an 8.2% increase in body weight on day 12 post-weaning in piglets receiving 3000 ppm ZnO compared to an unsupplemented control, which correlates with the 9.1% increase on day 15 post-weaning in the present study. Not only ZnO but also PFA 1 and PFA 2 supplementation improved the fecal scores of piglets between day 1 and 14 of the trial, with PFA 2 showing a greater effect than PFA 1. However, these observations were made under conditions with a low incidence of pathogens, as the overall mortality and medication rates as well as growth performance suggest a good health status of the whole group of animals observed in this study. The incidence of diarrhea, with a total of only 1.1% of all animals requiring medication due to PWD, was lower than the stated averages in the literature. The reported PWD rates in piglets receiving no ZnO or antibiotic supplementation are in the range of 34% to 51.1% for individual groups in Denmark [42,43] and between 3.6% and 14.3% in organized Indian farms [44]. The variations in the incidence of diarrhea and PWD might be the result of differing housing conditions, since, for example, low hygiene standards can lead to the increased susceptibility of piglets to stressors and diarrhea [3]. Due to the low overall morbidity of the piglets in this study, significant differences in health parameters like fecal scores are difficult to achieve, and the clinical relevance of the observed changes is up for discussion. Nevertheless, both ZnO- and carvacrol-containing feed additives have been demonstrated to improve piglet fecal scores in previous studies [41,45,46]. Therefore, it is assumed that also the differences found in the feeding trial are of clinical relevance.

The promising ability of tea tree oil to reduce the adherence of F4-fimbriated *E. coli* cells to piglets’ small intestinal mucus appeared to have only minor, if any, effects on fecal scores and the incidence of diarrhea in the presented feeding trial. However, since the reasons for the occurrence of PWD were not further explored, it cannot be specified whether the observed diarrhea events were caused by the F4-fimbriated *E. coli* strains in the present study. Notably, a subsequent study on PFA 2 was recently published [47]. In this study, the incorporation of PFA 2 in the diets of weaned piglets challenged with F4-Enterotoxigenic *E. coli* appeared to offer significant benefits in terms of reducing pathogen shedding and improving intestinal histomorphometry. This additional evidence strengthens the hypothesis on the mechanism through which PFA 2 interacts with pathogenic *E. coli* to support piglets during the post-weaning period and underscores the importance of continued research in this area.

Beneficial effects on growth parameters were observed for those substances that also led to improved fecal scores: The addition of PFA 2 to the feed resulted in a significant improvement of BW, ADG, and FCR, and thus performed close to ZnO. In contrast, supplementation with PFA 1 only improved FCR in a significant manner. These results reflect the in vitro observations, with PFA Core 2 showing effects at much lower concentrations compared to PFA Core 1 in the *E. coli* biofilm inhibition assay and the *C. violaceum* quorum sensing inhibition assay.

Blends containing carvacrol as one of their main constituents have previously been demonstrated to improve nutrient digestibility and the immunological as well as morphological gut health parameters of weaning piglets [45,46,48,49]. For example, dietary supplementation with a mixture of carvacrol and thymol reduced inflammatory responses by decreasing the expression of tumor necrosis factor α [45,48]. A similar mixture has also been shown to promote nutrient digestibility, enhance trypsin and chymotrypsin activity, and improve intestinal morphology via an increased villus height to crypt depth ratio [49]. The combination of all these effects may reduce the adverse effects of stress caused by weaning and bacterial infections, leading to the improved growth parameters observed in the present and other studies [45,46,49].

Differences in the application dosage may in turn explain the deviations between the present results and one of the few available studies on the use of tea tree oil as a feed additive, conducted by Wang et al. [50]. In contrast to the present study, these authors observed positive effects on the growth performance of weaned piglets at a tea tree oil dosage almost 15 times higher than the concentration used in our piglet study. This high amount of tea tree oil applied by Wang et al. is consistent with the concentrations that caused strong effects in the in vitro assays of the present study. This is a further indication that the substance concentrations that were necessary to achieve effects in our in vitro assays correlate with the substance doses required to improve the health status and growth performance of weaning piglets.

Similar to the present work, Grilli et al. [51] and Kelly et al. [52] successfully transferred results from in vitro assays to an in vivo context while investigating feed additives that reduce *Campylobacter jejuni* infectivity. However, it is important to note that most in vitro assays only investigate single aspects. These models usually lack the complexity of whole biological systems, such as the animal interphase or the microbiome, and thus simplify the in vivo situation. Nevertheless, a combination of various in vitro assays may serve as a useful toolbox for enhancing the understanding of the effects of phytogenics and predicting their effectiveness in vivo. Such an approach would increase the chances of success in formulating phytogenic feed additives to develop alternatives for traditional antimicrobial growth promoters, while minimizing the need for extensive animal trials.

## 5. Conclusions

The results of this study show that the dietary inclusion of a carvacrol–eugenol-star anise oil-based phytogenic feed additive improves growth parameters and fecal scores in weaned piglets compared to an unsupplemented control. While animal responses to feed additives can greatly vary, the growth performance improvement in this study was similar to, but less effective than, the addition of ZnO. The applied in vitro assays suggest that this effect could be associated with but not limited to the ability of carvacrol and eugenol to influence bacterial behavior and communication at the dosages included in the piglet study. Considering the in vitro results with tea tree oil, a single assay may not be sufficient to make predictions for these types of compounds. Taken together, this study has demonstrated that the applied in vitro models have the potential to support the development of phytogenic feed additives in order to find alternatives to traditional antimicrobial growth promoters in livestock feed while respecting the 3Rs principle. Particularly the use of a set of different in vitro assays with consistent results for prototype candidates can help to achieve this goal.

## Figures and Tables

**Table 1:** Piglet feed composition and calculated nutrients of starter and grower diets.

		Starter Diets	Grower Diets
		**Ingredients**	
Corn	%	27.84	26.44
Soybean meal (CP: 49%)	%	23.35	22.33
Barley	%	17.54	29.60
Wheat	%	12.91	13.70
Skim milk powder	%	10.00	-
Soybean oil	%	3.22	3.00
Limestone	%	1.44	1.56
Premix ^(1)^	%	1.20	1.20
Monocalcium phosphate	%	1.15	1.32
L-Lysine-HCL	%	0.50	0.30
DL-Methionine	%	0.20	0.10
L-Threonine	%	0.18	0.02
L-Tryptophan	%	0.07	0.03
Wheat bran	%	0.10	0.10
Tixosil ^(2)^	%	0.30	0.30
		**Calculated analysis**	
ME ^(3)^	MJ/kg	13.57	13.30
Crude protein	%	20.65	17.61
Lys	%	1.50	1.10
Met	%	0.55	0.37
Met + Cys	%	0.87	0.68
Thr	%	0.96	0.67
Trp	%	0.30	0.23
Crude fat	%	5.37	5.26
Crude fiber	%	3.47	4.12
Crude ash	%	5.96	5.71
Calcium	%	0.95	0.90
Available phosphorus	%	0.44	0.40
Sodium	%	0.22	0.20

^(1)^ Contents per kg premix: 400,000 IU vit. A (acetate); 120,000 IU vit. D_3_; 8000 mg vit. E (a-tocopherol acetate); 200 mg vit. K_3_ (MSB); 250 mg vit. B_1_ (mononitrate); 420 mg vit. B_2_ (cryst. riboflavin); 2500 mg niacin (niacinamide); 400 mg vit. B_6_ (HCl); 2000 mg vit. B_12_; 25,000 mg biotin (commercial, feed grade); 1000 mg pantothenic acid (Ca d-pantothenate); 100 mg folic acid (cryst. commercial feed grade); 80,000 mg choline (chloride); 5000 mg Fe (carbonate); NC, PFA 1, PFA 2: 5000 mg Zn (sulphate); 6000 mg Mn (sulphate); 1000 mg Cu (sulphate-pentahydrate); 20 mg Se (Na-selenite); 45 mg I (Ca-iodate); 130 g Na (NaCl); 55 g Mg (sulphate). ^(2)^ >97% silicon dioxide. ^(3)^ Metabolizable Energy, calculated as per DLG 2013.

**Table 2:** Minimum inhibitory concentrations of tested substances [ppm] against three *E. coli* strains and *C. violaceum*.

Strain	*C. violaceum*	*E. coli* O88:H8	*E. coli* O143:H4	F4+ *E. coli*
Individual substance				
Carvacrol	150	300	600	160
Eugenol	300	600	600	nt
Tea tree oil	2500	10,000	10,000	2560
Garlic oil	10,000	>10,000	>10,000	nt
Star anise oil	5000	>10,000	>10,000	nt
Zinc oxide	>10,000	>10,000	>10,000	nt
Individual substance				
PFA Core 1	2500	10,000	>10,000	nt
PFA Core 2	150	300	300	nt

nt = not tested.

**Table 3:** Effects of ZnO and phytogenic prototypes PFA 1 and PFA 2 on growth performance parameters of post-weaning piglets during the 42-day feeding trial.

Treatment	NC	ZnO	PFA1	PFA2	SEM	*p*-Value
						Treatment
BW day 1	7.06	7.08	7.07	7.05	0.198	1.000
BW day 15	11.34 ^a^	12.37 ^c^	11.65 ^ab^	11.90 ^b^	0.098	<0.0001
BW day 42	26.12 ^a^	28.52 ^c^	26.68 ^ab^	27.49 ^b^	0.228	<0.0001
ADG day 1–14	306 ^a^	378 ^c^	327 ^ab^	345 ^b^	6.936	<0.0001
ADG day 15–42	528 ^a^	577 ^c^	537 ^ab^	556 ^b^	6.012	<0.0001
ADG day 1–42	454 ^a^	511 ^c^	467 ^ab^	486 ^b^	5.464	<0.0001
ADFI day 1–14	356 ^a^	412 ^c^	367 ^ab^	381 ^b^	7.439	<0.0001
ADFI day 15–42	750 ^a^	780 ^b^	746 ^a^	760 ^ab^	7.900	0.014
ADFI day 1–42	619 ^a^	657 ^b^	619 ^a^	633 ^a^	6.538	<0.0001
FCR day 1–14	1.16 ^a^	1.09 ^c^	1.12 ^b^	1.10 ^bc^	0.008	<0.0001
FCR day 15–42	1.42 ^a^	1.35 ^c^	1.39 ^b^	1.37 ^bc^	0.008	<0.0001
FCR day 1–42	1.36 ^a^	1.29 ^c^	1.33 ^b^	1.30 ^bc^	0.007	<0.0001

Abbreviations: ZnO = Zinc oxide; PFA = Phytogenics feed additive; BW = body weight [kg]; ADG = average daily gain [g]; ADFI = average daily feed intake [g]; FCR = feed conversion ratio [g feed/g body weight gain]; SEM = Standard error of the means. Means bearing different superscript letters differ significantly with *p* < 0.05.

**Table 4:** Effects of ZnO and phytogenic prototypes PFA 1 and PFA 2 on fecal scores of post-weaning piglets during the 42-day feeding trial.

	NC	ZnO	PFA 1	PFA 2
**Starter feeding period: day 1 to day 14 on trial**				
Probability of normal feces ^(1)^	0.422 ^a^	0.821 ^d^	0.579 ^b^	0.681 ^c^
95% Confidence Interval	[0.352, 0.495]	[0.765, 0.866]	[0.505, 0.649]	[0.611, 0.743]
**Grower feeding period: day 15 to day 42 on trial**				
Probability of normal feces ^(1)^	0.870 ^a^	0.983 ^b^	0.855 ^a^	0.898 ^a^
95% Confidence Interval	[0.832, 0.900]	[0.969, 0.991]	[0.814, 0.888]	[0.865, 0.924]

^(1)^ Fecal scores were binarized as 0 = normal and 1 = altered (soft feces to severe diarrhea). Results are presented as the probability of normal feces. Means bearing different superscript letters differ significantly with *p* < 0.05.

## Data Availability

None of the data were deposited in an official repository. Data can be provided upon request via e-mail by the corresponding author.

## Supplementary Materials

The following supporting information can be downloaded at: https://www.mdpi.com/article/10.3390/ani15111661/s1, Figure S1: Effect of carvacrol on biofilm formation of *E. coli* strains O143:H4 and O88:H8 and violacein production of *C. violaceum*; Figure S2: Effect of eugenol on biofilm formation of *E. coli* strains O143:H4 and O88:H8 and violacein production of *C. violaceum*; Figure S3: Effect of garlic oil on biofilm formation of *E. coli* strains O143:H4 and O88:H8 and violacein production of *C. violaceum*; Figure S4: Effect of PFA Core 1 on biofilm formation of *E. coli* strains O143:H4 and O88:H8 and violacein production of *C. violaceum*; Figure S5: Effect of PFA Core 2 on biofilm formation of *E. coli* strains O143:H4 and O88:H8 and violacein production of *C. violaceum*; Figure S6: Effect of star anise oil on biofilm formation of *E. coli* strains O143:H4 and O88:H8 and violacein production of *C. violaceum*; Figure S7: Effect of tea tree oil on biofilm formation of *E. coli* strains O143:H4 and O88:H8 and violacein production of *C. violaceum*; Figure S8: Effect of ZnO on biofilm formation of *E. coli* strains O143:H4 and O88:H8 and violacein production of *C. violaceum*; Table S1: Mortality and causes of death from day 1 to day 42 on trial (day 25 to day 66 of age); Table S2: Clinical signs and medications from d 1 to d 42 on trial (d 25 to d 66 of age).

## Abbreviations

The following abbreviations are used in this manuscript:

ADFI	Average daily feed intake
ADG	Average daily gain
AGP	Antimicrobial growth promoter
BW	Body weight
diH_2_O	Deionized H_2_O
FCR	Feed conversion ratio
ME	Metabolizable energy
MIC	Minimum inhibitory concentration
NC	Negative control
PWD	Post-weaning diarrhea
TSB	Trypto-casein soy broth
ZnO	Zinc oxide
