# Supplementary material for: From Bench to Piglet: A Comparison of In Vivo and In Vitro Effects of Phytogenics on Post-Weaning Diarrhea, Growth Performance, and Bacterial Behavior
Source: Animals (Basel). 2025 Jun 4;15(11):1661. doi: 10.3390/ani15111661 (PMC12153809; doi:10.3390/ani15111661)
Supplement: Supplementary file 1 [file animals-15-01661-s001.zip › animals-3540136-SI.pdf]

From Bench to Piglet: A comparison of *in vivo* and *in vitro* effects of phytogenics on post-weaning diarrhea, growth performance, and bacterial behavior.

Anika Weitmann[a], Sonja Axmann[a], Klaus Männer[b], Teemu Rinttilä[c], Tobias Aumiller[d]

[a]*Institute for Animal Nutrition and Feed, AGES GmbH - Austrian Agency for Health and Food Safety, 4020 Linz, Austria*

[b]*Institute of Animal Nutrition, Department of Veterinary Medicine, Freie Universität Berlin, 14195 Berlin, Germany*

[c]*Alimetrics Research Ltd., Koskelontie 19B, FIN-02920 Espoo, Finland*

[d]*Delacon Biotechnik GmbH, 4209 Engerwitzdorf, Austria*

Corresponding author: Tobias Aumiller. Email: Tobias_Aumiller@cargill.com

***Animals* journal**

Supplementary material

A

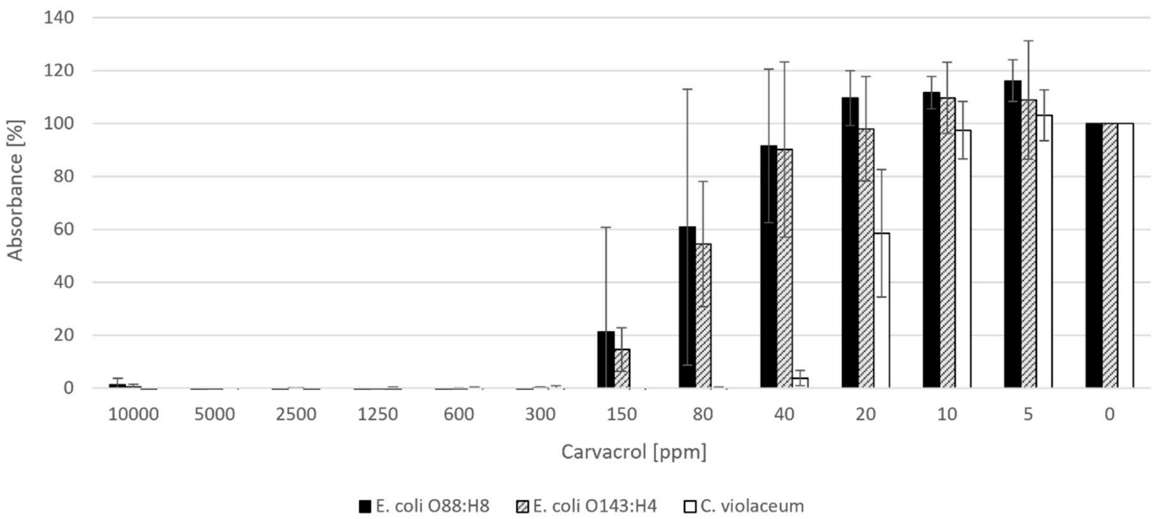

B

Carvacrol [ppm]	10 000	5 000	2 500	1 250	600	300	150	80	40	20	10	5	0
E. coli O88:H8	C	C	C	C	C	C	C	B	A	A	A	A	A
E. coli O143:H4	D	D	D	D	D	D	D	C	B	AB	A	A	AB
C. violaceum	C	C	C	C	C	C	C	C	C	B	A	A	A

Supplementary Figure S1. Effect of carvacrol on biofilm formation of *E. coli* strains O143:H4 and O88:H8 and violacein production of *C. violaceum*. (A) Results after 18 h incubation are presented as mean ± standard deviation of absorbance values, which were normalized to the positive control (growth without test substance). (B) Significant differences (*P*<0.05) between treatment concentrations within each bacterial model are indicated by different letters. Statistical analysis was performed using the glimmix procedure of SAS.

A

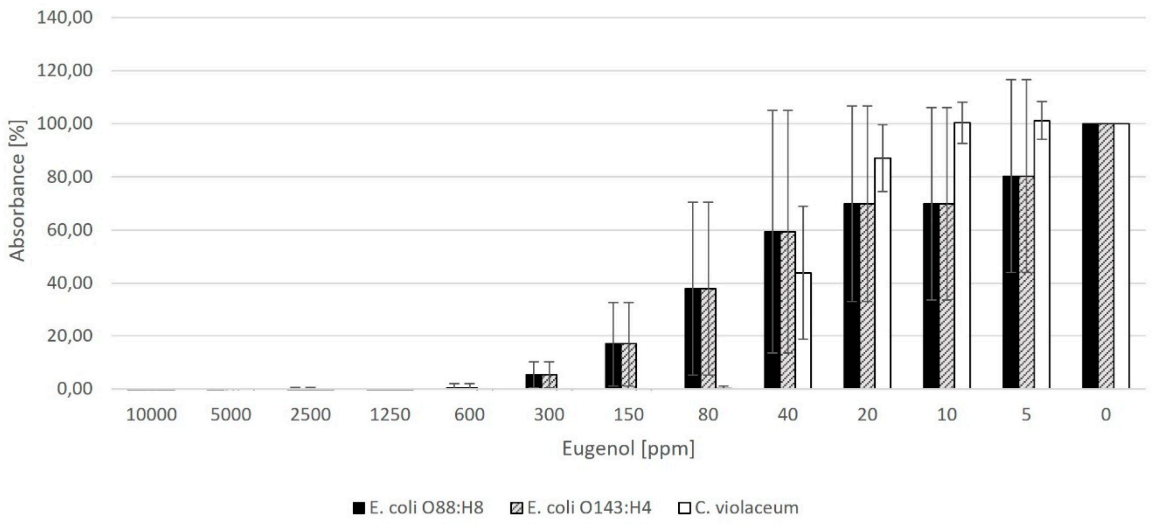

B

Eugenol [ppm]	10 000	5 000	2 500	1 250	600	300	150	80	40	20	10	5	0
E. coli O88:H8	E	E	E	E	E	DE	CD	BC	AB	AB	AB	A	A
E. coli O143:H4	E	E	E	E	E	E	DE	CD	BC	B	B	AB	A
C. violaceum	D	D	D	D	D	D	D	D	C	B	A	A	A

Supplementary Figure S2. Effect of eugenol on biofilm formation of *E. coli* strains O143:H4 and O88:H8 and violacein production of *C. violaceum*. (A) Results after 18 h incubation are presented as mean ± standard deviation of absorbance values, which were normalized to the positive control (growth without test substance). (B) Significant differences ($P<0.05$) between treatment concentrations within each bacterial model are indicated by different letters. Statistical analysis was performed using the glimmix procedure of SAS.

A

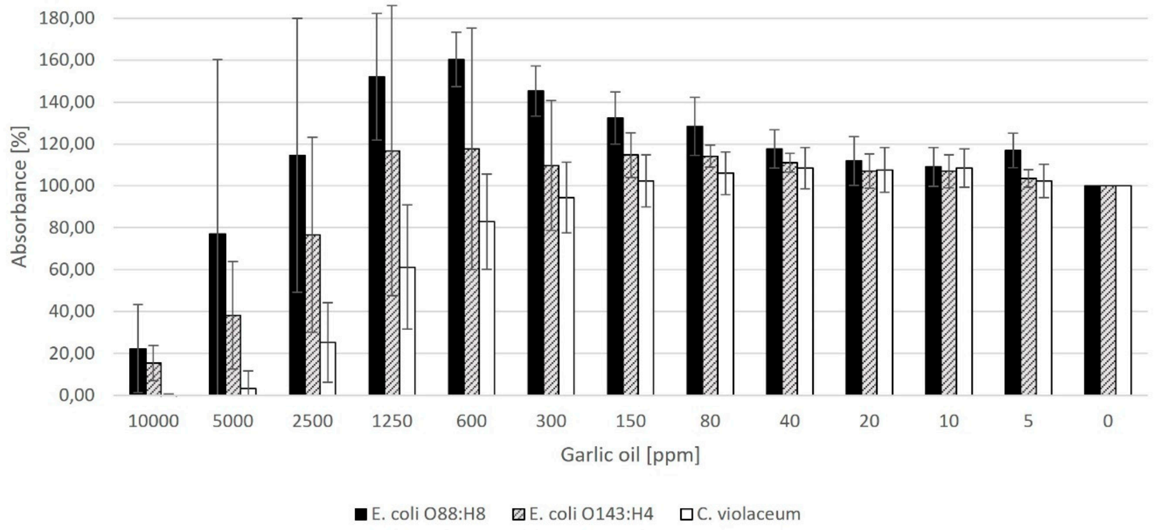

B

Garlic oil [ppm]	10 000	5 000	2 500	1 250	600	300	150	80	40	20	10	5	0
E. coli O88:H8	F	E	BCDE	AB	A	ABC	ABCD	ABCD	BCD	CDE	CDE	BCDE	DE
E. coli O143:H4	C	C	B	A	A	AB	A	A	A	AB	AB	AB	AB
C. violaceum	F	F	E	D	C	B	AB	A	A	A	A	AB	AB

Supplementary Figure S3. Effect of garlic oil on biofilm formation of *E. coli* strains O143:H4 and O88:H8 and violacein production of *C. violaceum*. (A) Results after 18 h incubation are presented as mean ± standard deviation of absorbance values, which were normalized to the positive control (growth without test substance). (B) Significant differences (P<0.05) between treatment concentrations within each bacterial model are indicated by different letters. Statistical analysis was performed using the glimmix procedure of SAS.

A

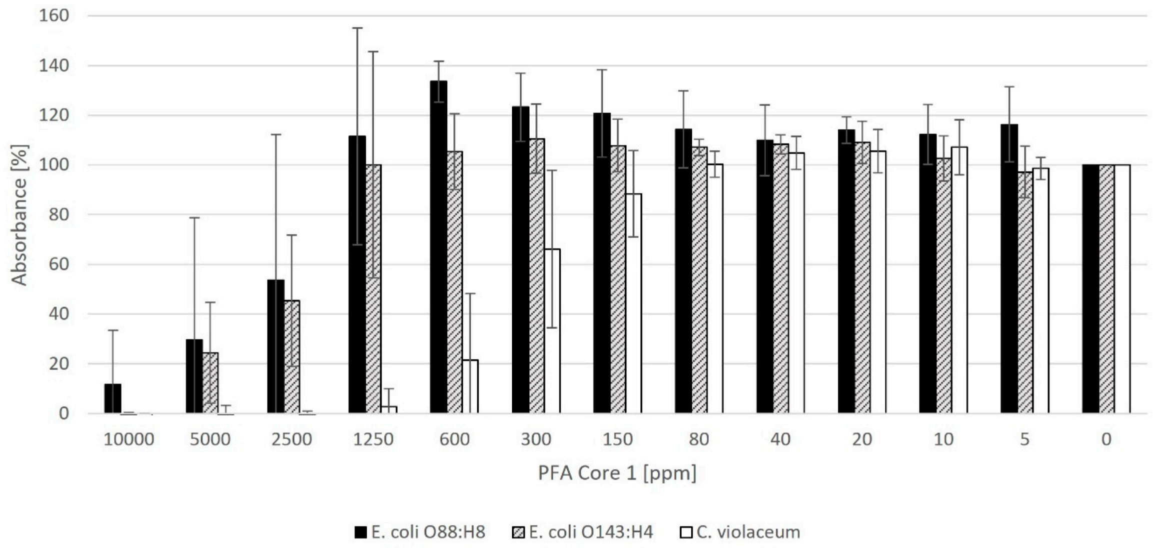

B

PFA Core 1 [ppm]	10 000	5 000	2 500	1 250	600	300	150	80	40	20	10	5	0
E. coli O88:H8	C	BC	B	A	A	A	A	A	A	A	A	A	A
E. coli O143:H4	C	B	B	A	A	A	A	A	A	A	A	A	A
C. violaceum	E	E	E	E	D	C	B	AB	A	A	A	AB	AB

Supplementary Figure S4. Effect of PFA Core 1 on biofilm formation of *E. coli* strains O143:H4 and O88:H8 and violacein production of *C. violaceum*. (A) Results after 18 h incubation are presented as mean ± standard deviation of absorbance values, which were normalized to the positive control (growth without test substance). (B) Significant differences (P<0.05) between treatment concentrations within each bacterial model are indicated by different letters. Statistical analysis was performed using the glimmix procedure of SAS.

A

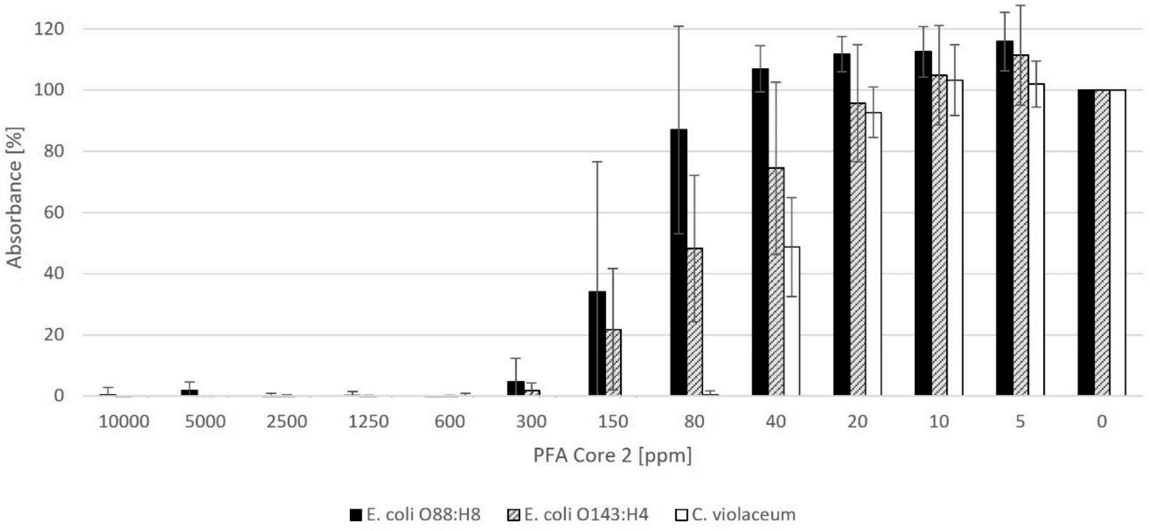

B

PFA Core 2 [ppm]	10 000	5 000	2 500	1 250	600	300	150	80	40	20	10	5	0
E. coli O88:H8	D	D	D	D	D	D	C	B	A	A	A	A	AB
E. coli O143:H4	F	F	F	F	F	F	E	D	C	B	AB	A	AB
C. violaceum	D	D	D	D	D	D	D	D	C	B	A	A	A

Supplementary Figure S5. Effect of PFA Core 2 on biofilm formation of *E. coli* strains O143:H4 and O88:H8 and violacein production of *C. violaceum*. (A) Results after 18 h incubation are presented as mean ± standard deviation of absorbance values, which were normalized to the positive control (growth without test substance). (B) Significant differences ($P<0.05$) between treatment concentrations within each bacterial model are indicated by different letters. Statistical analysis was performed using the glimmix procedure of SAS.

A

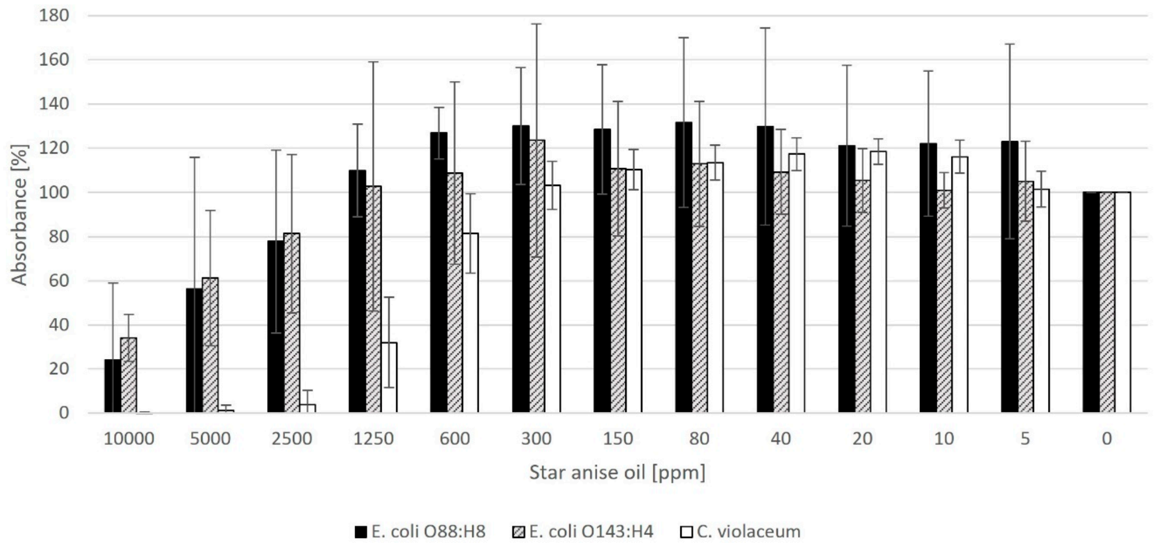

B

Star anise oil [ppm]	10 000	5 000	2 500	1 250	600	300	150	80	40	20	10	5	0
E. coli O88:H8	D	CD	BC	AB	A	A	A	A	A	AB	A	A	AB
E. coli O143:H4	D	CD	BC	AB	AB	A	A	A	AB	AB	AB	AB	AB
C. violaceum	F	F	F	E	D	BC	AB	A	A	A	A	BC	C

Supplementary Figure S6. Effect of star anise oil on biofilm formation of *E. coli* strains O143:H4 and O88:H8 and violacein production of *C. violaceum*. (A) Results after 18 h incubation are presented as mean ± standard deviation of absorbance values, which were normalized to the positive control (growth without test substance). (B) Significant differences (*P*<0.05) between treatment concentrations within each bacterial model are indicated by different letters. Statistical analysis was performed using the glimmix procedure of SAS.

A

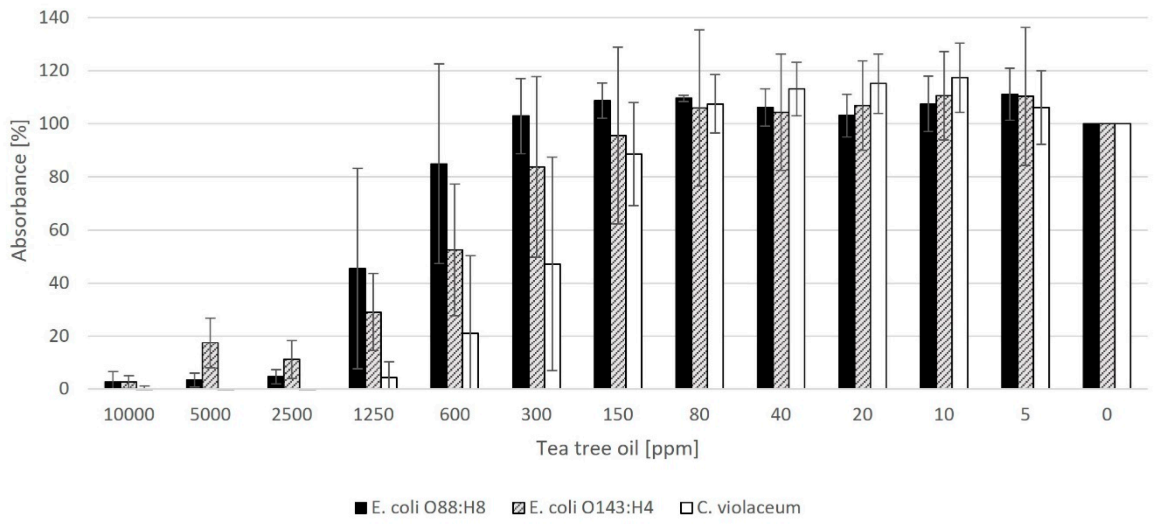

B

Tea tree oil [ppm]	10 000	5 000	2 500	1 250	600	300	150	80	40	20	10	5	0
E. coli O88:H8	D	D	D	C	B	AB	A	A	A	AB	A	A	AB
E. coli O143:H4	E	DE	E	D	C	B	AB	A	A	A	A	A	AB
C. violaceum	F	F	F	F	E	D	C	AB	AB	AB	A	AB	BC

Supplementary Figure S7. Effect of tea tree oil on biofilm formation of *E. coli* strains O143:H4 and O88:H8 and violacein production of *C. violaceum*. (A) Results after 18 h incubation are presented as mean ± standard deviation of absorbance values, which were normalized to the positive control (growth without test substance). (B) Significant differences ($P<0.05$) between treatment concentrations within each bacterial model are indicated by different letters. Statistical analysis was performed using the glimmix procedure of SAS.

A

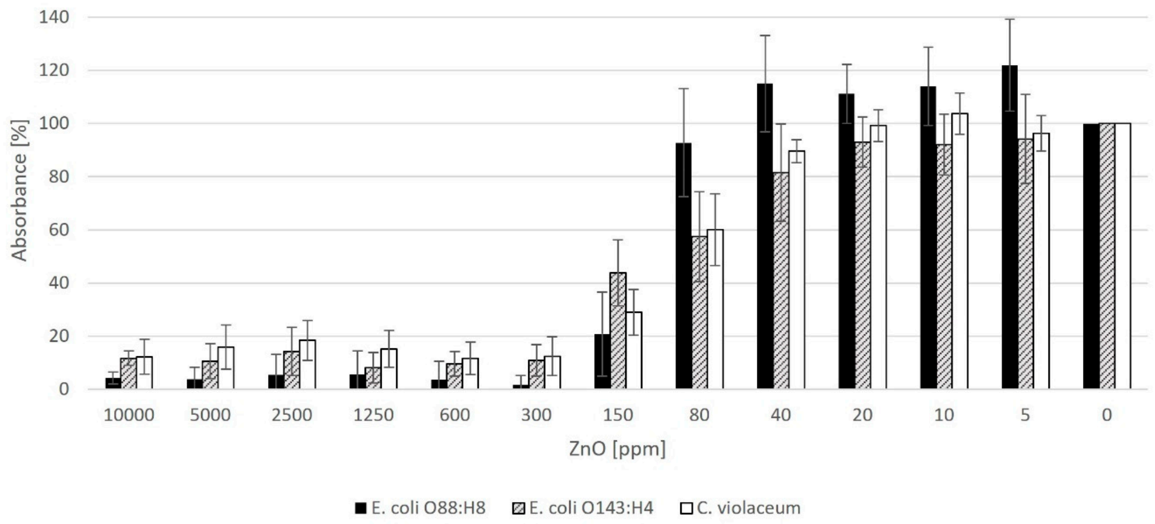

B

ZnO [ppm]	10 000	5 000	2 500	1 250	600	300	150	80	40	20	10	5	0
E. coli O88:H8	C	C	C	C	C	C	C	B	AB	AB	AB	A	AB
E. coli O143:H4	C	C	C	C	C	C	B	B	A	A	A	A	A
C. violaceum	E	E	E	E	E	E	D	C	B	A	A	AB	A

Supplementary Figure S8. Effect of ZnO on biofilm formation of *E. coli* strains O143:H4 and O88:H8 and violacein production of *C. violaceum*. (A) Results after 18 h incubation are presented as mean ± standard deviation of absorbance values, which were normalized to the positive control (growth without test substance). (B) Significant differences ($P<0.05$) between treatment concentrations within each bacterial model are indicated by different letters. Statistical analysis was performed using the glimmix procedure of SAS.

Supplementary Table S1: Mortality, and causes of death from day 1 to day 42 on trial (day 25 to day 66 of age)

Treatment / Pen n°	Age/days	Body weight	Cause
T1/11	28	6.31 kg	Colibacillosis, Pneumonia, Nephritis, Septicemia
T1/4	48	8.87 kg	Dermatitis, Septicemia, Nephritis
T1/18	56	13.11 kg	Colibacillosis, Peritonitis, Septicemia
T1/23	63	17.25 kg	Endocarditis, Pericarditis, Pneumonia
T2/12	49	12.93 kg	Pneumonia, Nephritis, Pericarditis
T3/19	41	09.63 kg	Pericarditis, Endocarditis, Pneumonia
T3/6	50	11.19 kg	Dilated Cardiomyopathy, Endocarditis, Pericarditis
T4/24	28	7.51 kg	Pneumonia, Septicemia

Supplementary Table S2: Clinical signs and medications from day 1 to day 42 on trial (day 25 to day 66 of age)

Treatment group	Number of piglets	Clinical signs
T1	16	Post-weaning diarrhea (6), Lameness (2); Respiratory Disorders (4), Exudative Dermatitis (4)
T2	14	Abscess (3); Respiratory Disorders (6), Exudative Dermatitis (5)
T3	18	Post-weaning diarrhea (3); Respiratory Disorders (5); Claw injuries (3), Exudative Dermatitis (7)
T4	13	Post-weaning diarrhea (2); Respiratory Disorders (6); Exudative Dermatitis (5)
% Piglets with medication: T1: 6.4%; T2 5.6%; T3: 7.2%; T4: 5.5%		

Baytril® (enrofloxacinum: 2.5 mg/kg/day) mainly in cases of post-weaning diarrhea; one-time application using intramuscular injection.
Hostamox® (amoxicillin: 15 mg/kg body weight/day) in cases of exudative dermatitis or respiratory and musculoskeletal disorders; three-time application using intramuscular injection every 24h.
Metacam® (meloxicam: 0.2 mg/kg body weight/day); one-time using intramuscular injection.
